# Recovery of Innate Immune Cells and Persisting Alterations in Adaptive Immunity in the Peripheral Blood of Convalescent Plasma Donors at Eight Months Post SARS-CoV-2 Infection

**DOI:** 10.3390/microorganisms9030546

**Published:** 2021-03-06

**Authors:** Ioannis V. Kostopoulos, Nikolaos Orologas-Stavrou, Pantelis Rousakis, Chrysanthi Panteli, Ioannis Ntanasis-Stathopoulos, Ioanna Charitaki, Eleni Korompoki, Maria Gavriatopoulou, Efstathios Kastritis, Ioannis P. Trougakos, Meletios-Athanasios Dimopoulos, Ourania E. Tsitsilonis, Evangelos Terpos

**Affiliations:** 1 Department of Biology, School of Science, National and Kapodistrian University of Athens, 15784 Athens, Greece; gikosto@gmail.com (I.V.K.); norologas@med.uoa.gr (N.O.-S.); rousakisp@gmail.com (P.R.); chrysanthipanteli23@gmail.com (C.P.); itrougakos@biol.uoa.gr (I.P.T.); rtsitsil@biol.uoa.gr (O.E.T.); 2 Department of Clinical Therapeutics, Alexandra General Hospital, School of Medicine, National and Kapodistrian University of Athens, 11528 Athens, Greece; johnntanasis@med.uoa.gr (I.N.-S.); j.charitaki@gmail.com (I.C.); e.korompoki@imperial.ac.uk (E.K.); mariagabria@gmail.com (M.G.); ekastritis@gmail.com (E.K.); mdimop@med.uoa.gr (M.-A.D.)

**Keywords:** SARS-CoV-2, COVID-19, convalescence plasma donors, immune profiling, immune restoration, 8 months

## Abstract

Persisting alterations and unique immune signatures have been previously detected in the peripheral blood of convalescent plasma (CP) donors at approximately two months after initial SARS-CoV-2 infection. This article presents the results on the sequential analysis of 47 CP donors at a median time of eight months (range 7.5–8.5 months) post infection, as assessed by flow cytometry. Interestingly, our results show a significant variation of the relevant immune subset composition among CP donors. Regarding innate immunity, both non-classical monocytes, and CD11b- granulocytes had fully recovered at eight months post COVID-19 infection. Intermediate monocytes and natural killer (NK) cells had already been restored at the two-month evaluation and remained stable. Regarding adaptive immunity, the COVID-19-related skewed Th1 and Th2 cell polarization remained at the same levels as in two months. However, low levels of total B cells were detected even after eight months from infection. A persisting reduction of CD8+ Tregs and changes in the NKT cell compartment were also remarkable. CP donors present with a unique immune landscape at eight months post COVID-19 infection, which is characterized by the notable restoration of the components of innate immunity along with a persisting imprint of SARS-CoV-2 in cells of the adaptive immunity.

## 1. Introduction

Coronavirus disease 2019 (COVID-19) is caused by the potentially fatal and highly contagious via airborne transmission severe acute respiratory syndrome coronavirus 2 (SARS-CoV-2). The severity of COVID-19 largely varies among infected people, ranging from cases entirely asymptomatic [1], to milder conditions experiencing fever, cough, and tiredness, and to more serious cases suffering from pneumonia leading to acute respiratory distress syndrome and unrestrained multi-organ failure, which are often fatal [2]. Symptomatic cases of COVID-19 are characterized by a systemic hyper-inflammatory immune response, and there is now ample evidence that variations in the severity of the symptoms following SARS-CoV-2 infection are directly related to the preceding host’s immune status [3,4,5]. Factors related to more dysfunctional immunocompromised immune profiles, and consequently, higher risk of mortality, including increased age and certain underlying chronic medical conditions, like type 2 diabetes, cardiomyopathy, obesity, chronic obstructive pulmonary disease, and chronic kidney disease [6,7].

Extensive research on COVID-19 has revealed important information on the immune cascades and particular changes in various immune subsets accompanying SARS-CoV-2 infection [3,4,8,9]. However, there is still limited information regarding the alterations that may persist in the host’s immune system over time after the complete resolution of the infection. Recently, it has been shown that distinct components of immunological memory to SARS-CoV-2 may have different kinetics over time [9,10]. For instance, anti-SARS-CoV-2 memory and cytotoxic T cells seem to decline more rapidly compared to B cell immunity, whereas both memory B cells and immunoglobulin (Ig)G levels against the spike protein of SARS-CoV-2 persist for at least six months post COVID-19 onset [10]. In a different cohort, Breton et al. showed that central memory CD4+ and CD8+ T cells decreased, but antigen-specific T cells persisted at six months after infection [11]. As for innate immunity cells, their profile seems to be age-dependent, and alterations in monocytes and neutrophils were observed up to ca. two months post infection [12]. We have also recently reported particular persisting alterations and unique immune signatures in the peripheral blood (PB) of convalescent plasma (CP) donors at approximately two months after initial SARS-CoV-2 infection. These immune signatures correlated both with the ability of patients to mount a protective humoral response, i.e., to produce sufficient and detectable anti-SARS-CoV-2 IgG and/or IgA antibodies, but also with the severity of the experienced symptoms, with previously hospitalized CP donors being less immunocompetent than CP donors who experienced milder symptoms [13]. Following the same approach and to explore for long-term imprints on the immune profile of COVID-19-recovered CP donors, we present here the results of the sequential analysis of 47 CP donors at a median time of eight months (range 7.5–8.5 months) post infection.

## 2. Materials and Methods

### 2.1. Selection of Participants

The present report includes sequential testing of CP donors who participated in a phase 2 study (NCT04408209) and provided paired PB samples to analyze their immune profile at two and eight months, post infection with SARS-CoV-2. All participants had been infected by SARS-CoV-2 and were diagnosed after positive nasopharyngeal and/or oropharyngeal swabs with RT-qPCR, as previously described [14]. Before the donation of CP, they had two negative PCR tests with an interval of at least seven days. The second negative test was performed 1–7 days prior to plasmapheresis. They also had developed detectable anti-SARS-CoV-2 IgG antibodies against the spike domain 1 (S1) protein of SARS-CoV-2 when examined with a sensitive commercially available, and Food and Drug Administration-approved Enzyme-linked ImmunosorbentAssay (Euroimmun Medizinische Labordiagnostika AG, Lubeck, Germany), described in detail previously [14]. Inclusion criteria also included an interval of at least 14 days after a complete recovery from SARS-CoV-2 infection (no symptoms, complete resolution of organ dysfunction which was caused by SARS-CoV-2), male donors without transfusion history and females without a history of transfusion or pregnancy, a normal complete blood count, negative serological tests for hepatitis B virus (HBV), hepatitis C virus (HCV), human immunodeficiency virus (HIV), venereal disease research laboratory (VDRL), human T cell lymphotropic virus (HTLV)-1 and negative for HIV, HBV, HCV with nucleic acid amplification testing, and donors fulfilled all the general criteria for blood donation in terms of age, general condition, hemoglobin levels, and vital signs.

Ten healthy individuals with similar age (median 47 years, range—45 to 73 years) and gender (6 male/4 female) were also evaluated and used as normal controls. These subjects had no known health problem and had a negative PCR test for SARS-CoV-2 from nasopharyngeal swabs at the time of blood sampling.

All participants were informed for the purposes of this study and provided signed informed consent according to the declaration of Helsinki and the local ethics committee. All study procedures were carried out in accordance with the declaration of Helsinki (18th World Medical Association Assembly), its subsequent amendments, the Greek regulation guidelines, as well as the Good Clinical Practice (GCP) guidelines defined by the International Conference of Harmonization. The protocol was approved by the Ethics committee of “Alexandra” General Hospital, Athens, Greece (Ref No 245/16 April 2020).

### 2.2. Flow Cytometry Analysis

PB samples were collected in EDTA-coated blood collection tubes (BD Vacutainer, BD Biosciences, San Jose, CA, USA; #367841) and were processed within an hour post collection following the Stain-Lyse-no-Wash protocol (BD Biosciences). In specific, for each PB sample, 100 μL of total anticoagulated blood were transferred to a 12 × 75 mm round-bottom polystyrene FACS tube and stained with a panel consisting of appropriate fluorophore-conjugated anti-human antibodies against the surface markers CD3-PerCP, CD4-BV510, CD8-PE, CD14-BV605, CD16-APC, CD25-PECF594, CD45-APCH7, CD56-APCR700, CD183-BV421, CD194-BV650, CD196-BB515 and CD11b-BV786 (all from BD Biosciences), at room temperature (RT) for 20 min protected from light. Further, 450 μL of 1× FACS Lysing Solution were added, and cells were incubated for another 10 min at RT protected from light. All samples were analyzed on a 3-laser BD FACSCelesta (BD Biosciences), and 100,000 events were acquired per sample.

The 12-color panel referred here consisted of our reference panel for the phenotypic analysis of CP donors at two months post infection and allowed for the identification and analysis of 24 distinct immune populations, including B cells, CD3+ T cells, natural killer (NK) cells, monocytes, granulocytes, and their subsets following the gating strategy previously described [13]. To be consistent with our previous batch analyses, we followed a regular and extensive set-up performance. The cytometer set-up was performed using unstained control cells for the configuration of the appropriate PMT voltages and with BD CompBeads Set anti-mouse Ιgκ (BD Biosciences) for the inter-channel fluorescence spillover compensation. Moreover, a CS&T daily performance was applied prior to each acquisition to verify the cytometer stable condition. Finally, an additional standardization step was applied daily using rainbow beads (Spherotech Inc, Lake Forest, IL, USA) to check for laser stability through the control of PMT fluctuations to detect the targeted fluorescent intensities of the beads.

### 2.3. Statistical Analysis

Data were analyzed using GraphPad Prism 8.0.2 software (San Diego, CA, USA). Results are expressed as means ± standard deviation (SD). For statistical analysis, a Student’s *t*-test was used to compare controls and CP donors eight months post SARS-CoV-2 infection, whereas changes in CP donors at two and eight months post infection were compared using paired Student’s *t*-test. *p*-values < 0.05 were considered statistically significant.

## 3. Results

### 3.1. Characteristics of CP Donors

The present analysis included 47 CP donors with paired PB samples collected at a median of two and eight (range 7.5–8.5) months post COVID-19 infection. The clinical characteristics of the participants are presented in Table 1.

### 3.2. The Immune Profiling at Eight Months Post SARS-CoV-2 Infection Does Not Correlate With the Clinical Characteristics of CP Donors

Consistent with our previous analysis at two months post SARS-CoV-2 infection [13], at eight months, we observed a significant variation of the relevant immune subset composition among CP donors. This variation exceeded the demographic and clinical characteristics of our cohort, as no significant differences could be associated with sex, age, the number of experienced symptoms, or the values of serum anti-SARS-CoV-2-specific antibodies against S1 viral antigen. Of note, no significant differences were obtained in immune profiling of CP donors who were previously hospitalized and those who had milder symptoms and did not meet the criteria for admission in intensive care units (ICUs) (data not shown). Therefore, we did not further subdivide CP donors into more categories in our subsequent analysis on the dynamics of immune profiling over time (Table 2).

### 3.3. Innate Immunity Recovery at Eight Months Post COVID-19

**Figure 1:**
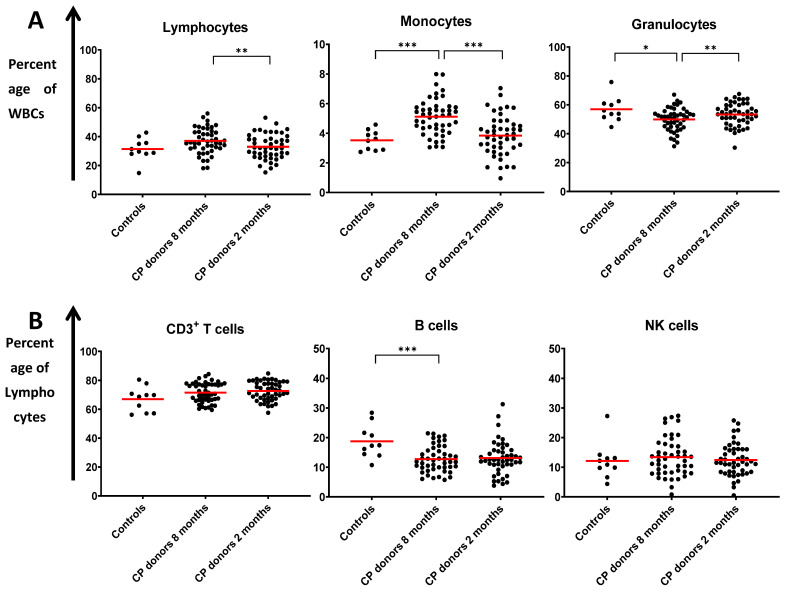
Distribution of (**A**) lymphocytes, monocytes, and granulocytes (expressed as a percentage of total white blood cells (WBC)) and (**B**) CD3+ T cells, B cells, and NK cells (expressed as a percentage of lymphocytes) in healthy donors (controls) and the two groups of recovered convalescent plasma (CP) donors at two and eight months post SARS-CoV-2 infection. Each point represents the percentage determined for each single donor. Red bars show mean values. *, *p* < 0.05; **, *p* < 0.01; ***, *p* < 0.001.

**Figure 2:**
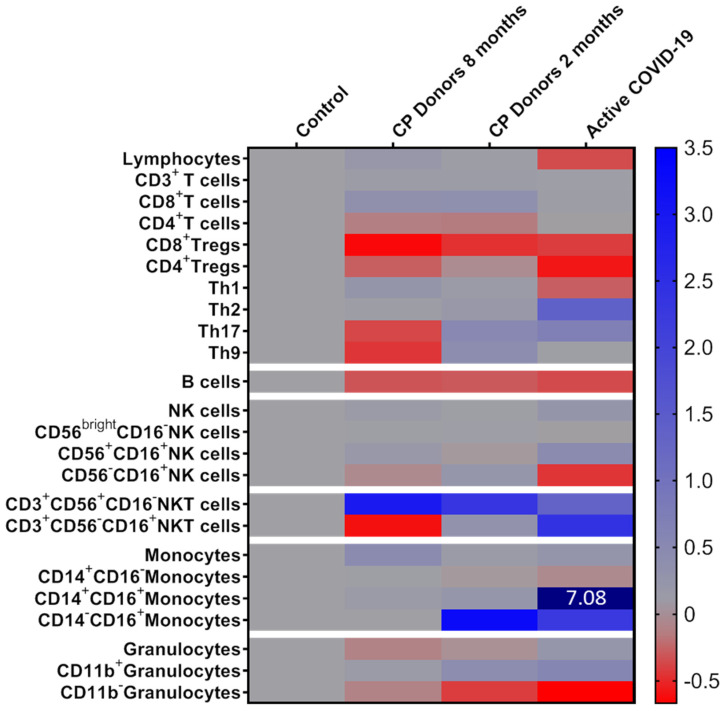
Heatmap of the mean percentages for white blood cell populations analyzed in recovered convalescent plasma (CP) donors at two and eight months, post infection and active COVID-19 patients, expressed as fold-increase compared to control group defined as 0. Colors were mapped linearly as in the colored key shown (low in red; high in blue). The numerical value indicates a difference exceeding the color scare bar limits.

We further assessed to evaluate the dynamics of particular immune profiles by comparing the relevant compositions of the various immune subsets over time. The analysis of the major immune populations belonging to the innate and the adaptive arms of immunity and their subpopulations are presented in Figure 1 and Figure 2. In general, our results show that by eight months post infection, the majority of innate immune cell subsets recovered to levels similar to those of the control group, contrarily to cells of the adaptive immunity, in which specific lymphocyte subsets showed evidence of persistent long-term activation.

Regarding monocyte subsets, we have previously reported a 7-fold increase of intermediate monocytes and a 2.2-fold increase of the non-classical compartment (at the expense of classical monocytes) during the active COVID-19 disease [11]. By two months post infection, the levels of intermediate and classical monocytes showed evidence of recovery, and had practically fully restored by eight months post infection. The percentages of non-classical monocytes, which remained increased at two months post infection (mean percentage—1.16% vs. 0.27% in the control group, *p* = 0.01), also fully recovered by eight-months and reached the levels of the control group.

Similar observations were recorded for granulocytes. Although their total percentage at eight months was slightly reduced compared to controls, at eight months post infection, the prevalence of the CD11b+ and CD11b- subsets have significantly decreased and increased, respectively, resulting in a CD11b+/CD11b- granulocyte ratio similar to that of normal healthy donors, as compared with the deregulated granulocyte expansion at the two-month evaluation [13].

As for NK cells, our analyses did not show any significant fluctuation between two and eight months, post infection. The percentages of total NK cells and their subsets almost restored at two months post active disease resolution, and the same profile was also seen at eight months, with percentages remaining at near-normal levels.

### 3.4. Alterations in Adaptive Immunity Persist at Eight Months Post Infection

In contrast to the innate arm of immunity, our data provide evidence of some persisting activation of the adaptive arm even at eight months post SARS-CoV-2 infection. It is noticeable that the percentage of total B cells did not restore to normal levels even after eight months (mean percentage—18.7% in controls vs. 13.1% and 12.7% in CP donors at two months and eight months post infection, *p* < 0.001), despite the fact that lymphopenia was fully reversed already at two months post infection. Regarding the CD4+ T cell compartment, our previous analysis highlighted an overall restoration of the COVID-19-related skewed CD4 polarization at two months post infection [13], except for some individual cases with a higher prevalence of the Th9- and Th17-type subsets. CP donors’ Th cells tested at eight months retained the recovery of the Th1/Th2 ratio, whereas percentages of Th17- and Th9-type cells decreased, sensibly due to the limited need for further activation of this type of polarized immune response. On the contrary, our analyses showed significant persisting changes at eight months post infection towards a decreased CD4/CD8 ratio (mean CD4/CD8 ratio—4.5 in controls vs. 2.2 at both two and eight months, post infection, *p* < 0.01) and a marked reduction of CD8+ Tregs that remained steadily low, since the initial infection. Furthermore, NKT cells and especially their CD56+CD16- compartment were found substantially increased even at eight months post COVID-19 infection. Of note, the percentages of the CD56+CD16- NKT compartment showed a significantly increased rate between the two- and the eight-month evaluation points (mean percentage—6% vs. 7.1% of total CD3+ cells at two and eight months, respectively, *p* = 0.002).

## 4. Discussion

COVID-19 is strongly associated with unique immune cascades, and several studies have reported the alterations in immune profiling following SARS-CoV-2 immune response [4,9,10,15]. However, the vast majority of these studies have focused on alterations during the active COVID-19 disease and/or within a short-term follow-up. In the present study, we report our results on the long-term evaluation of the immune profile of CP donors, who were previously infected with SARS-CoV-2 and mounted a humoral response, as confirmed by the production of specific antibodies against the S1-protein of the virus.

Our results show that some alterations, particularly in specific lymphocyte subsets of the adaptive arm of immunity, persist even at eight months post infection. We also noticed that at the same time point, the percentages of innate immunity major populations (monocytes, granulocytes, and NK cells), mostly of phenotypically distinct subsets thereof, were the same or very similar to the levels of healthy controls, suggesting that the resolution of the inflammatory profile is likely linked to the altered presence of these innate soluble-mediator (chemokine and cytokine)-producing cells.

Firstly, although the percentage of total monocytes in CP donors at eight months was increased compared to the two-month and control values, our data highlighted particular kinetics of the different monocytic subsets following COVID-19 infection. In more detail, we have previously reported a marginal increase of the total monocyte population in the PB of active COVID-19 patients, sensibly due to the generic recruitment of macrophages to the lung or other infected areas [13]. In agreement with other studies, we also noticed the apparent increase in the intermediate (CD14+CD16+) monocyte subset and a less significant elevation in the non-classical compartment (CD14-CD16+) at the expense of classical monocytes (CD14+CD16-) during the active COVID-19 phase [13,16,17]. The role of intermediate monocytes is not fully elucidated, though they are known to be antigen-presenting cells that participate in proliferation and stimulation of T cells with established involvement in anti-viral responses [18,19,20]. They are capable of secreting GM-CSF, TNF-α, and IL-6, which are critical for the inflammatory cytokine storms suffered by COVID-19 patients; hence, higher percentages of this subset have been associated with severe pulmonary complications, ICU admission, and increased time to discharge from the hospital [16,21]. The non-classical monocytes are mobile with a patrolling behavior in the endothelium, which may also be implicated in anti-viral responses [22]. Their unique phenotypic signature converges towards an anti-inflammatory mode of action, while new evidence supports their ability to undergo local conversion into alternatively activated macrophages [22,23]. In our cohort, we noticed that the percentages of intermediate monocytes recovered shortly (at two months) post infection, implying their critical role during the acute phase of COVID-19. On the contrary, the recovery of non-classical monocytes delayed and was evident at eight months, suggesting a prolonged immune activation lasting for at least two months post infection, further supporting their contribution to the resolution of inflammation. Our results are in agreement with Neeland et al., who most recently reported a similar monocyte signature in CP adults at seven weeks post infection, with increased percentages in total monocytes and a non-statistically significant increase in the same monocyte subsets as analyzed herein [12]. It should also be noted that in our analysis, total monocytes were gated on WBCs, and thus, may comprise phenotypes that do not necessarily express the markers used for identifying the aforementioned subsets (e.g., CD14- immature monocytes).

Interesting kinetics was also observed in granulocyte subsets which showed a gradual restoration over time, although the percentage of total granulocytes at eight months was slightly reduced compared to two-month values. As previously shown [13], the percentage of total granulocytes was increased during active COVID-19 with a substantial rise of their CD11b+ activated subset and a pronounced reduction of the CD11b- fraction. After a period of two months post infection, the percentages of CD11b+ granulocytes remained high in CP donors (mean—72.3% of total granulocytes in active COVID-19 vs. 64,5% in CP donors two months post infection vs. 46.1% in healthy donors), thus providing additional evidence of an ongoing immune response. At eight months post infection, the prevalence of the CD11b+ subset has significantly decreased, resulting in a CD11b+/CD11b- granulocyte ratio similar to that of normal healthy donors. CD11b, a protein subunit of the heterodimeric amb2, is a surface integrin present on several leukocytes that mediates among others cell migration, phagocytosis, chemotaxis, and cell-mediated cytotoxicity. CD11b immunophenotyping has been used to discriminate distinct immune profiles in lung inflammatory processes [24], whereas in COVID-19, we and others have highlighted the direct correlation between the increased CD11b+/CD11b- ratio to more adverse symptoms [13,25]. Moreover, we previously showed that these CP donors who did not develop SARS-CoV-2 antibodies despite their PCR positive testing had intermediate levels of CD11b+ granulocytes, lower compared to CP donors with detectable antibodies and comparable to unaffected controls, an observation that further associates the severity of the disease with hyperinflammation and stronger immune responses [26]. As our antibody panel did not include specific markers that would distinguish neutrophils from other granulocytes (eosinophils, basophils, mast cells), the reduction in their total percentage at eight months compared to two months could be due to lower percentages of these subsets in PB. In support, the number of eosinophils was shown to reduce during the CP phase [12], whereas activated mast cells that are implicated in the development of fibrotic conditions during active COVID-19, would also be expected to decrease after the resolution of the infection [27].

The percentages of NK cells at eight months were also similar to the levels of healthy controls. This is of importance since NK cells are crucial for anti-viral immunity and their effector functions are practically the first line of defense that limits viral replication. Thus, restoration of their levels to normal suggests a complete resolution of the infection at eight months. The marginal decrease in the CD56-CD16+ NK cell subset, a phenotype characterizing memory-like cells, additionally conforms with the resolution of the viral infection [28].

The aforementioned recovery of monocytes, granulocytes, and NK cells in the PB of COVID-19-recovered CP donors eight months post infection could be likely predicted. Viral clearance is largely a function of the innate immune system, being confronted with the rapid kinetics of virus elimination.

Nevertheless, profiling of the adaptive immune cells did not seem to follow the same pattern. We have previously reported significant alterations in adaptive cell subsets during the active COVID-19 phase, which quantitatively affected the percentages of B cells, T cells, and NKT cells due to the apparent lymphopenia that characterizes COVID-19, and accordingly affected the relevant composition of their various immune subsets. Firstly, the total number of B cells was found reduced throughout the whole study period and detected even at eight months post infection. Although our panel did not include specific B cell markers for an in-depth B cell subset analysis, the statistical significantly increased Th2-type cells during active COVID-19 combined with the reduced total numbers of B cells may imply a skewed ratio towards antibody-secreting effector B cells and maybe to terminally differentiated and highly secreting plasma cells [10]. The persisting deregulated B cell deficiency at eight months post COVID-19 infection may indicate B cell exhaustion in the periphery that needs more time to fully restore; it could also be a result of the hyper-inflammatory (IFN-γ high) environment during the active phase of COVID-19 reported to negatively impact on B cell development [29]. However, since CP donors included in the study developed detectable levels of anti-SARS-CoV-2 antibodies, a more detailed B cell panel is needed to clarify the reasons(s) of B cell impairment.

Convalescent individuals show a strong and probably durable memory immune response mediated by both CD4+ and CD8+ T cells [10,30,31]. In our study, the vast majority of CP donors showed increased percentages of CD8+ T cells both at two and eight months post infection, which, combined with the persisting low levels of CD8+ Tregs, probably indicate a durable cytotoxic immune reaction against possible SARS-CoV-2 residuals. A further support of this notion derives from the fact that NKT cells and especially the CD56+CD16- compartment were found substantially increased both at two months [13] and eight months post infection. CD3+CD56+ NKT cells share NK and T cell characteristics and reportedly have been considered as intermediate mediators of innate and adaptive immunity with an important role in anti-viral responses [32,33]. NKT cells, although found in very low numbers in PB, produce high levels of IFN-γ, regulate the equilibrium of Th1/Th2 cells, and enhance CD4+ and CD8+ T cell functions, whereas when depleted in vivo, viral replication is greatly enhanced. In COVID-19, increased levels of NKT cells were noticed mostly in mild cases, and were proposed to promote rapid control of the infection, being either directly cytotoxic or mediating antibody-dependent cell-mediated cytotoxic effects [34]. Therefore, the presence of notable percentages of NKT cells up to eight months post infection may, on the one hand, support the evidence that the inflammatory trigger (i.e., likely some inactive viral remnants) is not fully cleared, but on the other hand, may also indicate an NKT cell-mediated cytotoxic activation that is still alert and probably compensates the role of the restored to-nearly-normal levels NK and CD8+ T cells.

Regarding the limitations of our study, we should note that the evaluation of the components of immune response against SARS-CoV-2 was among the secondary study endpoints in the context of a phase 2 clinical trial of CP. Thus, it may be underpowered in terms of highlighting significant associations in subgroup analyses. Furthermore, all the analyses were performed in PB samples from the participants. Since COVID-19 is primarily a pulmonary disease, alveolar samples could provide a more accurate overview of the immune responses generated.

## 5. Conclusions

In conclusion, CP donors present with a unique immune landscape at eight months post COVID-19 infection, which is characterized by the restoration of the components of innate immunity along with a persisting imprint of SARS-CoV-2 on the adaptive immunity. Elucidating the short- and long-term immune cell alterations to SARS-CoV-2 is essential in terms of therapeutics, prevention, and for formulating effective vaccination strategies. Although the long-term immune response following vaccination remains to be determined, the sustained alterations of adaptive immunity following COVID-19 seem to reinforce the value of vaccination. Vaccines aim to prime the immune system against SARS-CoV-2 more rapidly than natural infection. Neutralizing antibodies and SARS-CoV-2-specific memory B and T cell responses will subsequently prevent the infection or at least control the infection in a timely manner in order to prevent severe disease.

## Figures and Tables

**Table 1:** Demographic and clinical characteristics of CP donors included in this study.

Clinical Characteristics	CP Donors (*n* = 47)
Male gender (N/percentage)	27 (57.4%)
Age in years, median (range)	49 (37–72)
**Symptoms (N/percentage)**	
Fever	12 (25.5%)
Fatigue	15 (31.9%)
Headache	27 (57.4%)
Cough	26 (55.3%)
Breathlessness	35 (74.5%)
Diarrhea	34 (72.3%)
Anosmia	22 (46.8%)
Ageusia	25 (53.2%)
Anti-S1 IgG, median OD (IQR)	5.14 (6.23)

CP, convalescent plasma; OD, optical density; IQR, interquartile range.

**Table 2:** Mean percentages of immune cell populations with the relative statistical significance of differences between groups.

Immune Subset	Controls N = 10	CP Donors Eight Months N = 47	CP Donors Two Months N = 47	*p* Value **	*p* Value ***
Lymphocytes (% of WBCs)	31.41 ± 7.78 *	36.95 ± 8.74	32.96 ± 8.57	ns	0.002
CD3^+^ T Cells (% of Lymphocytes)	67.07 ± 8.61	71.51 ± 6.80	72.70 ± 6.50	ns	ns
CD8^+^ T Cells (% of CD3^+^ T Cells)	24.38 ± 8.51	32.65 ± 9.37	33.38 ± 10.38	0.013	ns
CD4^+^ T Cells (% of CD3^+^ T Cells)	71.36 ± 11.28	61.40 ± 10.12	60.43 ± 10.82	0.008	ns
CD8^+^ Tregs (% of CD8^+^ T Cells)	7.29 ± 8.24	2.67 ± 3.74	3.86 ± 4.95	0.011	ns
CD4^+^ Tregs (% of CD4^+^ T Cells)	2.04 ± 0.90	1.48 ± 0.90	1.87 ± 1.28	ns	<0.001
Th1 (% of CD4^+^ T Cells)	26.77 ± 7.96	32.71 ± 10.35	29.25 ± 10.32	ns	0.022
Th2 (% of CD4^+^ T Cells)	7.67 ± 3.60	8.13 ± 9.11	8.86 ± 14.71	ns	ns
Th17 (% of CD4^+^ T Cells)	1.73 ± 0.96	1.09 ± 1.79	2.61 ± 6.63	ns	ns
Th9 (% of CD4^+^ T Cells)	11.57 ± 4.10	6.45 ± 6.01	16.15 ± 16.74	0.013	<0.001
B Cells (% of Lymphocytes)	18.74 ± 5.60	12.74 ± 4.39	13.13 ± 5.59	<0.001	ns
NK Cells (% of Lymphocytes)	12.14 ± 6.13	13.41 ± 6.43	12.43 ± 5.42	ns	ns
Immature NK Cells (% of NK Cells)	0.69 ± 0.32	0.71 ± 0.63	0.72 ± 0.63	ns	ns
Mature NK Cells (% of NK Cells)	8.84 ± 5.05	10.33 ± 5.53	8.55 ± 4.84	ns	0.001
Memory-like NK Cells (% of NK Cells)	2.61 ± 1.20	2.37 ± 1.48	3.16 ± 1.81	ns	0.007
CD3^+^CD56^−^CD16^+^ NKT Cells (% of CD3^+^ Cells)	0.19 ± 0.13	0.07 ± 0.15	0.25 ± 0.47	0.029	0.014
CD3^+^CD56^+^CD16^−^ NKT Cells (% of CD3^+^ Cells)	1.80 ± 2.05	7.07 ± 6.13	6.00 ± 5.29	0.010	0.002
Monocytes (% of WBCs)	3.52 ± 0.66	5.11 ± 1.20	3.84 ± 1.34	<0.001	<0.001
Classical Monocytes (% of Monocytes)	95.04 ± 3.14	97.57 ± 2.13	91.95 ± 12.22	0.003	<0.001
Intermediate Monocytes (% of Monocytes)	0.81 ± 0.64	0.89 ± 1.14	0.99 ± 1.21	ns	ns
Non-classical Monocytes (% of Monocytes)	0.27 ± 0.24	0.27 ± 0.54	1.16 ± 2.45	ns	0.012
Granulocytes (% of WBCs)	56.84 ± 8.56	49.86 ± 7.80	53.20 ± 7.88	0.014	0.002
CD11b^+^ Granulocytes (% of Granulocytes)	46.12 ± 34.14	50.94 ± 23.16	64.52 ± 25.10	ns	0.001
CD11b^−^ Granulocytes (% of Granulocytes)	47.29 ± 27.52	41.43 ± 18.13	27.38 ± 18.47	ns	<0.001

* mean percentage ± standard deviation; ** statistical significance of controls vs. CP donors at eight months (Student’s *t*-test); *** statistical significance of CP donors at eight months vs. CP donors at two months (paired Student’s *t*-test); ns, non-significant.

## Data Availability

Data available upon request from the authors.
